# The Role of Population Receptive Field Sizes in Higher-Order Visual Dysfunction

**DOI:** 10.1007/s11910-024-01375-6

**Published:** 2024-09-12

**Authors:** Deena Elul, Netta Levin

**Affiliations:** 1 fMRI Unit, Neurology Department Hadassah Medical Organization, Faculty of Medicine, The Hebrew University of Jerusalem, POB 12000, Jerusalem, 91120 Israel; 2 Edmond and Lily Safra Center for Brain Sciences (ELSC), The Hebrew University of Jerusalem, Jerusalem, Israel

**Keywords:** Population receptive field, Functional MRI, Visual cortex, Hemianopia, Spatial integration

## Abstract

**Purpose of Review:**

Population receptive field (pRF) modeling is an fMRI technique used to retinotopically map visual cortex, with pRF size characterizing the degree of spatial integration. In clinical populations, most pRF mapping research has focused on damage to visual system inputs. Herein, we highlight recent work using pRF modeling to study high-level visual dysfunctions.

**Recent Findings:**

Larger pRF sizes, indicating coarser spatial processing, were observed in homonymous visual field deficits, aging, and autism spectrum disorder. Smaller pRF sizes, indicating finer processing, were observed in Alzheimer’s disease and schizophrenia. In posterior cortical atrophy, a unique pattern was found in which pRF size changes depended on eccentricity.

**Summary:**

Changes to pRF properties were observed in clinical populations, even in high-order impairments, explaining visual behavior. These pRF changes likely stem from altered interactions between brain regions. Furthermore, some studies suggested that pRF sizes change as part of cortical reorganization, and they can point towards future prognosis.

## Introduction

**Fig. 1:**
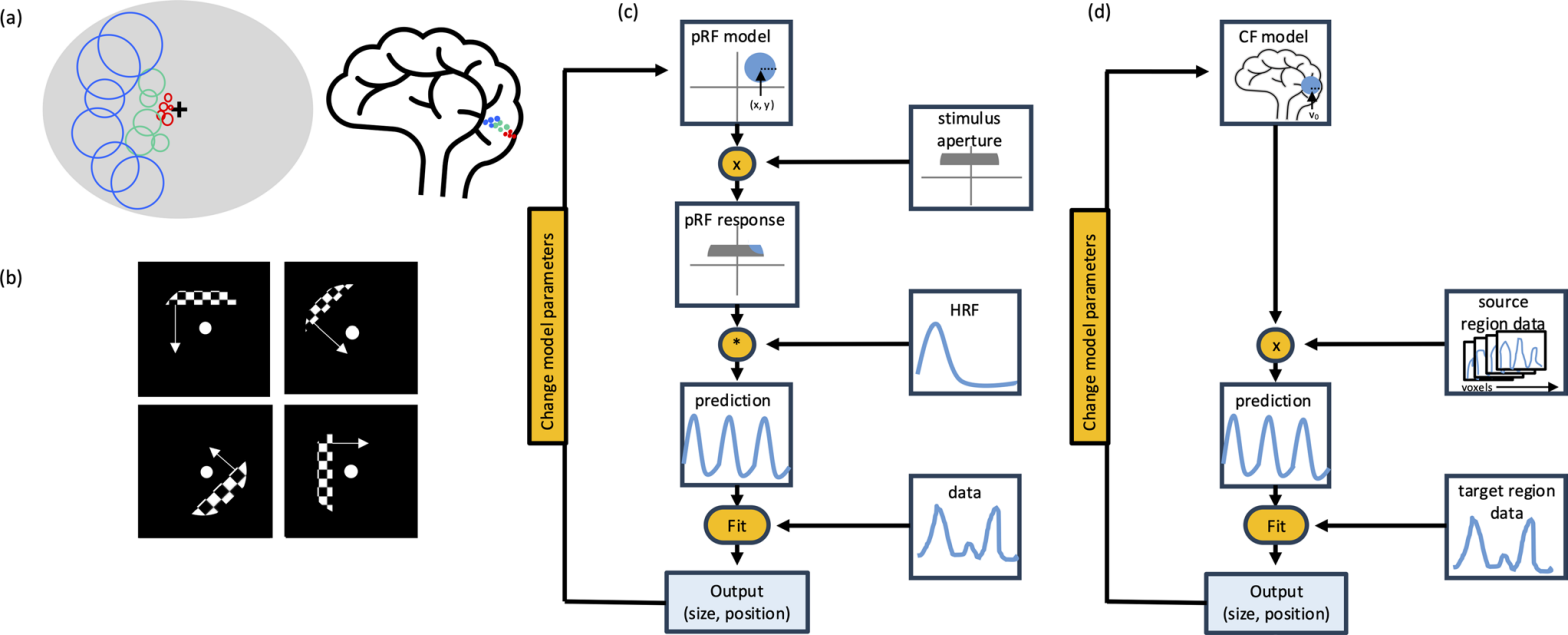
Population receptive field (pRF) and Connective field (CF) modeling procedures. **a**) Diagram of population receptive fields shown on the visual field and along the calcarine sulcus **b**) Example stimuli used for pRF and CF mapping **c**) Diagram of the pRF model fitting procedure (adapted from [4]) **d**) Diagram of the CF model fitting procedure (adapted from [11])

Vision is a perceptual experience in which information is received by the retina and processed in a series of hierarchical areas in the cortex, going through a complex process of encoding, abstraction, and interpretation. At a basic level, the visual system is organized according to two different principles. One level of organization is topographic: each point in the visual field is mapped to a specific point in visual cortex. In a topographic map, known in the visual system as a retinotopic map, adjacent points in the visual field are mapped to adjacent points in cortex (Fig. 1a). An additional level of organization is functional: some visual areas respond to specific stimulus categories in a selective manner. For example, the fusiform face area responds to faces more than other stimulus categories [1]. Retinotopic organization is characteristic of low-level visual areas which are not functionally specialized (do not respond in a selective manner to specific stimulus categories) [2]. However, in recent years, studies have found that functionally specialized high-level areas are also organized retinotopically [3].

Population receptive field (pRF) modeling is a functional MRI (fMRI) technique used for mapping retinotopic organization in visual areas. Blood-oxygen-level-dependent (BOLD) responses are measured in response to a stimulus, typically a checkerboard bar, that moves across the visual field (Fig. 1b). These responses are used to fit a model of the population receptive field of each voxel (averaged over thousands of neurons). Simple pRF analyses model each receptive field as a Gaussian with position and size parameters, representing the location and extent in visual space where the voxel responds to visual stimulation [4] ( Fig. 1c). More recent pRF models capture additional aspects of neuronal receptive fields and visual responses. Difference-of-Gaussian (DoG) models represent surround inhibition with an inner excitatory Gaussian and an outer inhibitory Gaussian [5], compressive spatial summation models capture nonlinearities in spatial responses [6], and divisive normalization models aim for a more biologically plausible representation [7]. pRFs have also been identified in high-level visual areas, including face- [8], motion- [9], place- [8], and word-selective [10] regions.

Another extension of pRF modeling is connective field (CF) modeling (Fig. 1d). CF modeling characterizes information transmission throughout the visual system by predicting activity in one cortical area as a function of activity in another area. This can be used to assess convergence of visual information. For example, if connective fields from V1 to a single voxel in V2 are larger than connective fields from V2 to a single voxel in V1, this indicates that information converges moving up the hierarchy; in other words, there is increased spatial integration [11].

pRF mapping can be used to delineate different regions of early visual cortex by separating their retinotopic maps. pRF eccentricity and polar angle parameters are computed from the fitted position parameters, and retinotopic areas are demarcated based on polar angle and eccentricity shifts. pRF mapping can also be used to calculate visual field coverage, or the portion of the visual field which responds to visual stimulation. This is done by taking all voxels which are significantly fit by the pRF model, then combining their size and position parameters to create a map of visual field locations that evoke cortical activity.

Prior work has found that pRFs within each retinotopic map increase in size and eccentricity as their position progresses from posterior to anterior calcarine. Additionally, pRF sizes become progressively larger from one map to the next throughout the visual hierarchy [4]. pRF size is an important metric for studying spatial integration in visual areas. Smaller pRF sizes, typically found in the fovea, allow high-resolution processing [12]. Larger pRF sizes allow more extensive integration over space, as is necessary for processing high-level stimuli such as faces [13].

Previous studies have looked at retinotopic mapping in the visual cortex following damage to the visual system. Most of these focused on conditions affecting the eye, including the lens [14], cornea [15], retina [16, 17], photoreceptors [18], and optic chiasm [19, 20]. Some of these studies investigated not just how pRF properties change with damage, but also how they can reflect recovery from damage or, in other words, cortical reorganization. For example, prior studies looked at pRF properties following sight recovery [14, 15] or recovery of cone photoreceptor function [18].

Fewer studies have looked at pRF properties in the visual cortex following damage to the brain itself. Here, we highlight recent research on the role of pRF modeling in high-level visual dysfunctions, focusing on primary visual cortex and beyond. We suggest that in clinical populations, the properties of pRFs in the visual system can explain complex visual impairments.

### **Population** R**eceptive** Field Properties Following Visual Field Deficits

Recent research has investigated pRF properties in patients with homonymous visual field deficits (HVDs) due to damage beyond the optic chiasm. HVDs most often emerge following stroke [21] and can interfere with visual performance and daily activities [22]. It is well established that many patients with V1 lesions retain the ability to respond unconsciously to visual information in visual field locations where conscious perception is impaired, a phenomenon known as blindsight [23, 24]. The cortical pathways involved in blindsight are a subject of debate. Blindsight could potentially be mediated by subcortical pathways that bypass V1 and transmit information to undamaged extrastriate areas such as the hV5/MT + complex, which is involved in motion perception [25, 26]. Alternatively, it could be facilitated by undamaged “islands” in the V1 blind field [27]. It is also unclear to what degree blindsight depends on cortical reorganization. Some patients exhibiting blindsight retain visually evoked BOLD responses in V1 [28, 29] and/or extrastriate areas [28, 30–34], but the relationship between cortical activity and visual performance is unclear.

Several studies have used pRF modeling to probe the cortical basis of blindsight, exploring whether visually responsive voxels remain in the blind field and whether patients undergo cortical reorganization. Papanikolaou et al. [35] measured V1 pRF properties in five patients with quadrantanopia, finding that pRF coverage maps did not necessarily agree with behavioral perimetry maps. In three patients, visually responsive voxels overlapped with the scotoma, indicating the presence of spared islands in V1. In two patients, conscious vision was found in visual field locations lacking V1 responses, suggesting the presence of V1-bypassing pathways. More recently, the same group tested pRF properties in the hV5/MT + complex [36]. hV5/MT + has been linked to blindsight, as prior studies have found evidence that it responds to moving stimuli presented within the scotoma following V1 lesions [31–34, 37]. All patients exhibited BOLD responses in hV5/MT + within the blind field. In two patients, BOLD responses covered visual field locations that were not covered by V1, indicating pathways that bypass early visual areas [36].

In terms of pRF size, Papanikolau et al. [35] found that some quadrantanopia patients exhibited larger pRF sizes than controls in V1 of the lesioned hemisphere near the scotoma border, as well as in the contra-lesional hemisphere. Barbot et al. [38] recently replicated these findings, testing 11 patients with HVDs and finding that pRFs for voxels in the blind field were more eccentric and larger than for voxels in regions where conscious vision was retained. Changes in the lesioned hemisphere could reflect reduced inhibition near the scotoma border, while changes in the contra-lesional hemisphere may stem from disrupted interhemispheric input [35]. In hV5/MT+, three patients exhibited larger pRF sizes than controls, in both the lesioned and the contra-lesional hemispheres. However, one patient exhibited smaller pRF sizes in the contra-lesional hemisphere in hV5/MT+ [36]. Thus, specific effects may vary based on individual subjects’ lesion characteristics, making it difficult to draw firm conclusions.

Two recent studies have looked at pRF changes in the context of visual restitution training (VRT), a therapy for HVDs which involves repetitive presentation of stimuli within the blind field or along its border. It is important to note that VRT protocols have elicited much controversy, in part because they are not well-validated [39] and because benefits are variable across patients [40]. Elshout et al. [41] tested 40 patients with HVDs resulting from stroke, with no control or placebo group. The authors mapped the visual field using both perimetry and pRF techniques, then repeated the perimetry measurements following VRT. Barbot et al. [38] tested 11 patients with HVDs and age-matched controls, conducting both perimetry and pRF measurements before and after VRT. Notably, both studies mapped the visual field using Humphrey perimetry, which Leitner et al. argued is not sufficiently accurate to measure changes in visual field coverage [42].

Elshout et al. [41] found that VRT elicited the largest behavioral improvements in parts of the visual field which evoked cortical responses prior to training. Similarly, Barbot et al. [38] found that coherence and strength of visually evoked responses in blind field voxels near the scotoma border before training predicted the amount of visual field recovery following VRT. Furthermore, increased V1 coverage of the blind field and larger pRF sizes in V1 blind field voxels, particularly at higher eccentricities, were found following the treatment. The authors argued that increased pRF sizes could be explained by repeated attentional deployment to the trained visual field locations during VRT [38]. These two studies suggest that the presence of cortical responses, but not behavioral responses, in particular visual field locations before training could indicate increased plastic potential, making these locations more effective candidates for behavioral improvement with training [41].

### Population Receptive Field Properties Following Hemispherectomy

In very rare cases, hemispherectomy can be used to treat intractable epilepsy in pediatric patients [43]. A few case reports have used pRF modeling to analyze visual cortical organization in these patients. Haak et al. [44] studied a patient whose entire left hemisphere was functionally removed at age three to treat Rasmussen syndrome, leading to a complete right HVD. In the lateral occipital cortex (LOC) of the remaining hemisphere, they identified an atypical visual field map characterized by small pRF sizes and an overrepresentation of low eccentricities. The authors noted that LOC typically contains large receptive fields which span both sides of the visual field, and smaller pRF sizes are consistent with the disruption of input from one hemisphere. More recently, Halbertsma et al. [45] replicated the pRF analysis in the same patient in a wider range of visual areas. In both earlier and later visual areas, the patient’s pRF sizes were smaller than controls’ at lower eccentricities, but within the normal range at higher eccentricities. Additionally, the patient’s visual field maps were foveally biased in later visual areas, replicating prior results [44]. Georgy et al. [46] tested a different patient who underwent a functional right hemispherectomy at age 17. While maps in the patient’s left hemisphere retained mostly normal organization, pRF sizes were significantly larger than controls’ in V2d and V3d for eccentricities greater than 4°. The differences between patients could stem from variation in the causes, circumstances, or age of cortical damage. It is also worth noting that pRF sizes can vary at least twofold even among subjects with normal vision [4, 12], making it difficult to draw conclusions given the small number of subjects tested.

In their case study, Halbertsma et al. [45] also fit bilateral pRF models in V1, representing each pRF as two Gaussians mirrored around either the horizontal or the vertical meridian. The presence of bilateral pRFs would indicate that early visual cortex reorganizes following hemispherectomy, representing information from both sides of the visual field instead of just one side. However, bilateral pRF models were not superior to the single pRF model in the patient tested, indicating that cortical reorganization was limited. In contrast, bilateral pRFs have been reported in patients with a congenitally nonfunctioning hemisphere [47]. Differences between these two types of patients helps illuminate critical periods in cortical organization and development.

### Population Receptive Field Properties in Aging and Alzheimer’s Disease

Aging is associated with a decline in low-level visual functions such as acuity [48], spatial contrast sensitivity [49], motion perception [50], and processing speed [51]. These changes are potentially related to loss of retinal ganglion cells, which occurs during aging [52] and has been associated with increased receptive field sizes in a glaucoma model [53]. However, the relationship between these factors is unknown.

In four normally aging subjects and five young adults, Brewer and Barton [54, 55] found age-related changes in the central 3° of V1, V2, and hV4, including lower proportional surface area and larger pRF sizes in older subjects. These changes, which are specific to foveal representations, could explain reduced acuity in aging.

To explore potential mechanisms behind these changes, Silva et al. [56] conducted tests of visual acuity, pRF properties, and retinal structure in 50 subjects aged 20–80. They found that older age was associated with larger pRF size in V1, V2, and V3, and that the increase in pRF sizes with age was less pronounced moving up the visual hierarchy. Additionally, larger pRF size was negatively correlated with visual acuity (in V1-V3) and visual field map surface area (in V1-V2). (The correlation between pRF size and surface area had been previous demonstrated in V1 [12]). Retinal thickness measurements also decreased with age, and they were correlated with V1-V3 surface area but not visual acuity or pRF sizes. However, since all measures were strongly correlated with age, it is difficult to infer directional effects. One possible interpretation is that pRF sizes increase due to deterioration of retinal ganglion cells and their ascending projections, which leads to shrinking of V1 and disrupted cortical dynamics.

pRF mapping could potentially be used for early detection of cortical changes associated with Alzheimer’s disease, since a variety of low- and high-level visual deficits can appear as early symptoms [57]. As a proof of concept, Brewer and Barton [55] conducted pRF mapping in two patients with early-stage mild Alzheimer’s disease. These two subjects exhibited irregularities in visual field map size and organization, and both had smaller pRF sizes in relatively peripheral regions of V2, V3, and hV4. While this experiment demonstrates the feasibility of such measurements, more detailed tests in a larger subject pool are needed to characterize pRF changes associated with Alzheimer’s disease.

### Population Receptive Field Properties in Posterior Cortical Atrophy

Patients with posterior cortical atrophy (PCA), a rare variant of Alzheimer’s disease characterized by deterioration of high-level visual areas, often exhibit foveal crowding, in which nearby stimuli disrupt image recognition, as well as simultanagnosia, in which objects and scenes appear fragmented [58]. These two symptoms support opposite predictions about pRF size. Foveal crowding is consistent with larger pRF sizes, which could disrupt high-resolution processing by pooling multiple images in the same receptive field [58, 59]. Simultanagnosia is consistent with smaller pRF sizes, which could disrupt spatial integration [13].

pRF mapping experiment in five patients with PCA [60] revealed larger pRF sizes relative to controls in V1 and hV4 at low eccentricities, consistent with foveal crowding. At high eccentricities, smaller pRF sizes relative to controls were found in V1, hV4, and the temporal occipital regions TO1/2 (corresponding to hV5/MT+ [9]), which could explain simultanagnosia. A similar pattern of results was observed when analyzing pRF size changes with eccentricity: the typical increase in pRF size with eccentricity was reduced or even reversed in patients’ V1 and hV4. Behavioral experiments using a masked repetition-priming task revealed a reduced fovea-to-periphery processing gradient in patients, consistent with larger foveal pRF sizes. However, group differences were not observed in intermediate visual areas.

The observed differences in pRF size in early visual cortex could be caused by disrupted feedback connections and impaired attentional processes following atrophy of high-level visual cortex [60]. To test this possibility, connective field (CF) modeling was conducted in six PCA patients, exploring information transmission and convergence between different areas in visual cortex. In the CF modeling experiment, convergence did not increase along the visual hierarchy in PCA patients as it did in controls, particularly in the dorsal stream. This indicates that in PCA patients, higher-order dorsal stream areas such as TO1/2 may receive input from a more spatially restricted area in V1, which could explain simultanagnosia. In contrast, V3d appeared to sample from a larger V1 region in patients than in controls, perhaps reflecting a compensatory mechanism [61].

Overall, these results suggest that PCA-related atrophy in high-level visual areas can lead to receptive field size changes in low-level areas as well, and that these changes in early areas can explain high-order visual deficits [60].

### Population Receptive Field Properties in Autism Spectrum Disorder

Many individuals with autism spectrum disorder (ASD) exhibit differences in perceptual behavior, including enhanced local processing at the expense of global processing [62–65]. This local bias could lead one to expect that ASD individuals have reduced pRF sizes. Schwarzkopf et al. [66] conducted a pRF mapping experiment in 15 individuals diagnosed with ASD and 12 neurotypical controls. In contrast to expectations, they found larger pRF sizes in ASD individuals in several extrastriate areas, including V2, V3, and V4, particularly at perifoveal eccentricities. In V3, where the group difference was largest, pRF sizes were correlated with individual differences in autistic traits as measured by the autism-spectrum quotient (AQ). No significant differences were observed in V1 or V3A.

Schwarzkopf et al. [66] also observed larger pRF sizes in MT+, which is associated with motion perception [26], although this did not survive correction for multiple comparisons. In a behavioral experiment, Schauder et al. [67] found that motion perception was impaired among ASD individuals relative to controls for small stimuli, regardless of contrast. This was consistent with a model of larger excitatory receptive fields in ASD, which impair perception of stimuli much smaller than the receptive fields.

These results suggest that enhanced local processing in ASD is not driven by smaller receptive fields or sharper spatial selectivity. Schwarzkopf et al. [66] offered two alternative explanations for the behavioral and cortical differences. Firstly, hyper-responsiveness of visual cortex in ASD (due to changes in excitation/inhibition balance or regulation) could lead to larger BOLD responses to stimuli outside the pRF center, resulting in larger estimated pRF sizes. Alternatively, individuals with ASD could employ a different attentional style where they focus more on the center of gaze, leading to a blurring of peripheral representations and consequently larger pRF sizes.

### Population Receptive Field Properties in Schizophrenia

Schizophrenia is associated with atypical visual perception in various tasks, especially those which involve visual context or integration. Patients experience reduced disruption [68] or facilitation [69] from visual context, and they exhibit impairments in global processing [70], orientation discrimination [71], contrast sensitivity [72], and motion processing [73]. Anderson et al. [74] conducted retinotopic mapping in V1-V4 for 18 schizophrenia patients, finding that patients had smaller pRF sizes in various visual areas for both 2D Gaussian and difference-of-Gaussians (DoG) models. In the DoG model, schizophrenia patients exhibited pRFs with smaller inhibitory surrounds in V1, V2, and V4, as well as smaller excitatory centers in V1 and V2. Smaller inhibitory surrounds are consistent with theories of reduced surround suppression in schizophrenia, which can explain many of the observed perceptual differences.

## Conclusions

fMRI-based pRF modeling allows us not only to map the visual cortex but also, through estimation of pRF size, to characterize the degree of spatial integration. Different vision-related cortical pathologies are accompanied by changes in pRF parameters (see Table 1 for summary). Larger pRFs, indicating coarser spatial processing, were observed in HVDs [35, 36, 38], aging [54–56], and ASD [66]. Smaller pRFs, indicating finer processing, were observed in Alzheimer’s disease [55] and schizophrenia [74]. For conditions such as posterior cortical atrophy, pRF size changes depended on eccentricity: pRFs were smaller for high eccentricities and larger for low ones [60].

**Table 1:** Summary of pRF size results

Condition	Relevant papers	Number of patients	pRF size change	Eccentricity of pRF size change	Brain area of pRF size change	Potential explanations
Homonymous visual field deficits	Papanikolaou et al., 2014 [35] Papanikolaou et al., 2019 [36] Barbot et al., 2021 [38]	5 [35, 36] 11 [38]	Increase	Near blind field border, especially low eccentricities [38]	V1 [35,^38^] hV5/MT+ [36]	Decreased inhibition around the lesion [35]; reorganization of inter-hemispheric connections [36]; increased excitability [38]; increased plastic potential [38]; differences in effective contributions of neuronal subpopulations [38]; changes in balance of bottom up/top-down inputs [38]; loss of neural resolution [38]; disorganization of neural positions [38] Increases in pRF size with training may result from repeated attentional deployment [38]
Hemispherectomy	Haak et al., 2014 [44] Halbertsma et al., 2019 [45]	1	Decrease	Lower eccentricities	V1, V2, V3, V3A, LO1, LO2, hV4 [45]	Loss of input from opposing hemisphere [44]; disrupted cortical development [44]; development of a preferred retinal locus as an adaptation to HVD [45]
	Georgy et al., 2019 [46]	1	Increase	Higher eccentricities	V2d, V3d	Loss of input from subcortical areas or opposing hemisphere
Aging	Brewer & Barton, 2012, 2014 [54, 55] Silva et al., 2021 [56]	4 [54, 55] 50 (all ages) [56]	Increase	Lower eccentricities	V1, V2, V3, hV4	Loss of retinal ganglion cells and their axons, leading to a smaller V1 map, disrupted neural interactions, and larger pRF sizes [56]
Alzheimer’s disease	Brewer & Barton, 2014 [55]	2	Decrease	Higher eccentricities	V2, V3, hV4	Not specified
Posterior cortical atrophy	de Best et al., 2019 [60]	5	Increase	Lower eccentricities	V1, hV4, TO	Atrophy of high-level visual cortex, leading to disrupted feedback connections and impaired attentional processes
			Decrease	Higher eccentricities		
Autism spectrum disorder	Schwarzkopf et al., 2014 [66]	15	Increase	Higher eccentricities	V2, V3, V4	Hyper-responsiveness of visual cortex; changes in attentional style
Schizophrenia	Anderson et al., 2017 [74]	18	Decrease	Not specified	V1, V2, V4	Changes in excitation/inhibition balance leading to reduced surround suppression

**Fig. 2:**
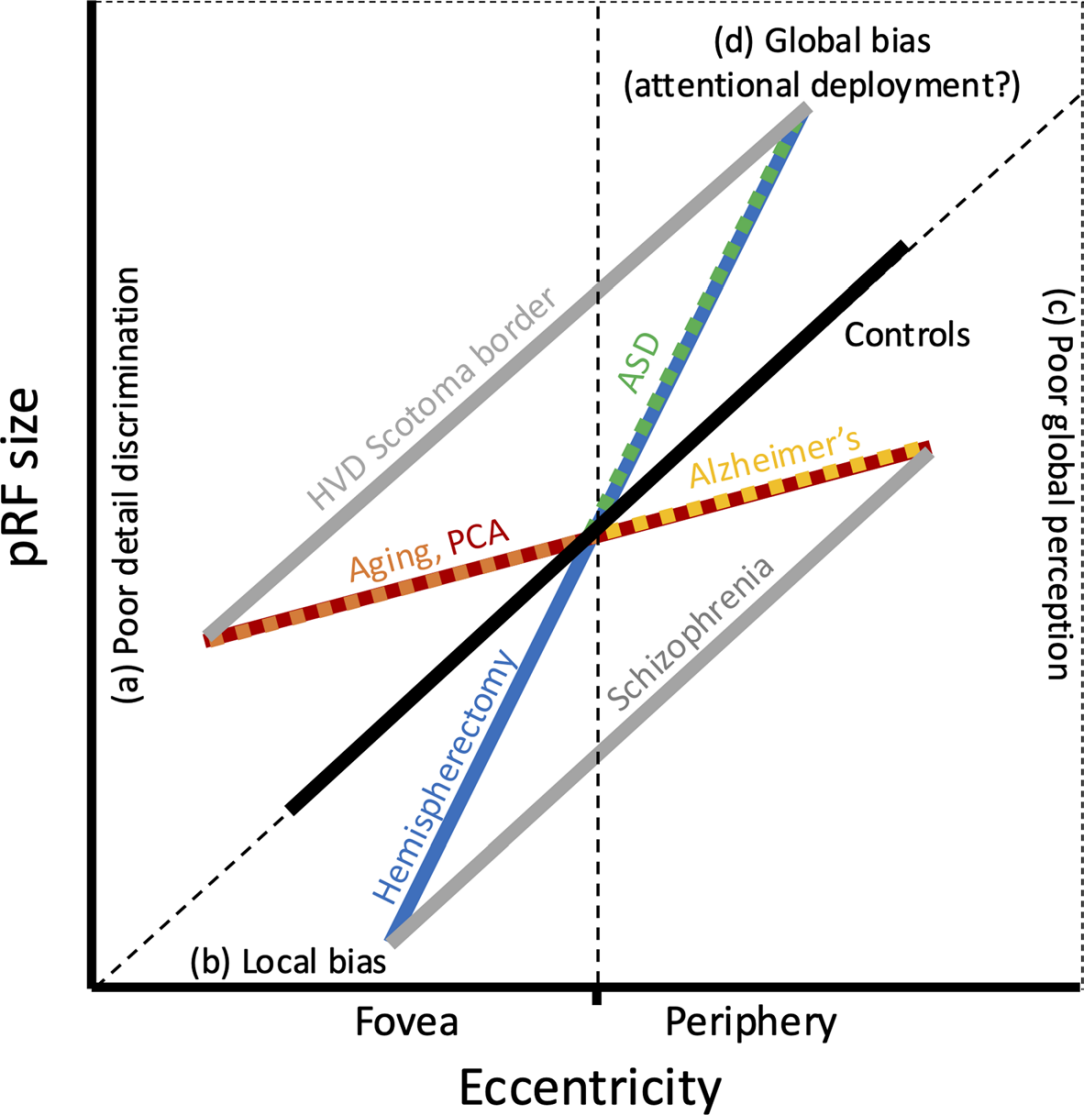
Graphical summary of population receptive field (pRF) size results and suggested interpretation. Patient groups’ pRF sizes divided into four quadrants according to their deviation from controls’ pRF sizes (foveal/peripheral eccentricities, smaller/larger than controls’). **a**) Larger pRF sizes in the fovea may reflect poor detail discrimination. **b**) Smaller pRF sizes in the fovea may reflect local bias. **c**) Smaller pRF sizes in the periphery may reflect poor global perception. **d**) Larger pRF sizes in the periphery may reflect a global processing bias or changes in attentional deployment

Changes in pRF sizes have different behavioral implications depending on whether they occur in the fovea or the periphery (see Fig. 2 for summary). Larger pRF sizes in the fovea, seen in HVDs [35, 36, 38], aging [54–56], and PCA [60], indicate difficulty processing detail, such as reduced acuity and foveal crowding (Fig. 2a). Smaller foveal pRF sizes, as seen in schizophrenia [71] and hemispherectomy [44, 45], indicate a local processing bias (Fig. 2b). Smaller peripheral pRF sizes, observed in Alzheimer’s [55], schizophrenia [74], and PCA [60], indicate global perceptual deficits, such as simultanagnosia and visual integration difficulties (Fig. 2c). Larger peripheral pRFs, as seen in HVDs [35, 36, 38], hemispherectomy [46], and ASD [66], suggest a global processing bias, or they may reflect eccentricity-dependent changes to attentional processes (Fig. 2d).

It is important to note that many of the studies we reviewed involved very few patients with pathologies that are not uniform, leading to sporadic results. Despite this, we want to offer some insights that emerge from the review and make suggestions for future applications.

### What is the Basis of Population Receptive Field Size Changes?

In most of the studies reported above, the observed changes in pRF size were attributed to changes in the relationships between different brain areas, whether adjacent areas, distant regions within the same hemisphere, or areas in different hemispheres.

In HVDs, increased pRF sizes near the scotoma border were suggested to result from reduced surround inhibition around the lesion [35], while increased pRF sizes in the contra-lesional hemisphere and in hV4/MT + were suggested to result from disruption to interhemispheric connections [36]. Other disease models suggest even more distant effects, even at the level of visual input. For example, in aging, the loss of ganglion cells and their axons could disrupt neuronal interactions, leading to larger pRF sizes [56].

Reduced pRF sizes could also stem from disrupted feedback connections and changes in excitation/inhibition balance. In the case of hemispherectomy, smaller pRF sizes were suggested to stem from the loss of input from the contralateral hemisphere [44]. However, smaller pRF sizes, which usually reflect finer spatial processing, may also indicate cortical reorganization. In the case of hemispherectomy, for instance, it was suggested that smaller pRF sizes reflect the development of a preferred retinal locus as an adaptation to central vision loss [45].

As the reader can see, the mechanisms suggested in the reviewed papers for reduced pRF sizes overlap with the mechanisms suggested for increased pRF sizes. We suggest that due to the brain’s complex balance of feedforward and feedback connections, similar changes in connectivity or excitation/inhibition balance could theoretically lead to either increased or reduced pRF sizes, depending on the specific brain regions and connections involved. Indeed, for some conditions, particularly neurodegenerative diseases, the observed pattern of changes in pRF sizes depended on eccentricity, sometimes with increased and decreased pRF sizes observed within the same patients. In PCA, larger pRF sizes were observed in the fovea, while smaller pRF sizes were observed in the periphery [60]. Additionally, in aging subjects, increases in pRF sizes were most prominent in the fovea, consistent with the reduction in visual acuity that occurs with age [54, 55]. pRF size changes in autism spectrum disorder [66] and Alzheimer’s disease [55] were restricted to higher eccentricities. These eccentricity-specific changes support the notion that parts of visual cortex that encode different eccentricities receive feedback connections from different parts of the brain [75].

### What Can pRF Size Tell us About Current Behavior and Future Outcomes?

Several studies suggest that pRF mapping could be used as a tool for developing targeted treatments for high-level visual deficits. Elshout et al. found that in patients with HVDs, visual field locations which were covered by pRFs were more likely to recover with visual restitution training [41]. Similarly, Barbot et al. found that visually evoked responses in blind field voxels near the scotoma border before training predicted the amount of visual field recovery following VRT [38]. These results suggest that pRF mapping could be used to identify patients who are good candidates for VRT, as well as regions within patients’ visual fields where perception could improve with training. This could allow the development of training protocols that are targeted to individual patients’ visual and cortical deficits, potentially yielding improved outcomes.

A couple recent studies investigated the connection between pRF properties and behavior. In PCA patients, a masked repetition-priming task revealed a reduced fovea-to-periphery processing gradient, consistent with the observation of larger foveal pRF sizes [60]. Additionally, following Schwarzkopf et al.’s [66] observation that pRF sizes in extrastriate areas are increased in ASD, Schauder at al. [67] conducted psychophysical experiments which revealed motion processing deficits consistent with larger pRF sizes. Investigations of this type could help determine when changes in pRF properties are clinically significant and how they affect visual behavior and performance. Future research is needed to explore the connection between pRF properties and behavior for additional conditions affecting the visual system.

Research on pRF mapping in clinical populations is still in its early stages. Given the rare occurrence of some of the conditions, many studies have tested very small samples. In some cases, such as hemispherectomy, conflicting results were found in different patients [44–46], making it difficult to draw firm conclusions. Differences between patients likely stem from specific lesion characteristics. Since the brain is a complex system involving both feedforward and feedback connections, damage in one area can affect many other areas, and small differences in the type and location of brain damage can lead to substantially different effects. This stands in contrast to damage to inputs of the visual system, which lead to more predictable results. To account for heterogeneity between individuals, future work could focus on testing larger patient samples.

## Data Availability

No datasets were generated or analysed during the current study.
